# Esophageal leiomyoma and simultaneous overlying squamous cell carcinoma: a case report and review of the literature

**DOI:** 10.1186/s12893-021-01214-2

**Published:** 2021-04-29

**Authors:** Saadat Mehrabi, Mohammad Javad Yavari Barhaghtalab, Safoora Hejazinia, Hossein Saedi

**Affiliations:** 1 Department of General Surgery, Shahid Beheshti Hospital, Yasuj University of Medical Sciences, Yasuj, Iran; 2 Department of Pathology, Shahid Beheshti Hospital, Yasuj University of Medical Sciences, Yasuj, Iran; 3 Department of Anesthesiology, Shahid Beheshti Hospital, Yasuj University of Medical Sciences, Yasuj, Iran

**Keywords:** Esophageal squamous cell carcinoma, Leiomyoma of esophagus, Simultaneous occurrence, Overlying, Esophageal resection, Case report

## Abstract

**Background:**

Squamous cell carcinoma is the most common epithelial tumor of the esophagus. Upper endoscopy with multiple minimally invasive biopsies should be performed to confirm the diagnosis. Leiomyoma of esophagus is rare, but it’s the most common benign submucosal mesenchymal tumor of the esophagus. The simultaneous occurrence of an overlying epithelial lesion and a mesenchymal lesion is very rare. This study aims to show a case operated due to squamous cell carcinoma of esophagus that was postoperatively diagnosed with coexistent esophageal leiomyoma and give a clear overview of the existing literature on it.

**Case presentation:**

The patient was a 41-year-old woman who underwent three field esophagectomy (McKeown). Pathological evaluation was done, and the patient had poorly differentiated squamous cell carcinoma and multiple leiomyomas. A leiomyoma was found with an invading overlying squamous cell carcinoma.

**Conclusion:**

It is concluded that esophageal carcinomas may coexist with leiomyomas; preexisting benign tumors may have played an important role in the development of the carcinoma by inducing constant stimulation of the overlying mucosa; endoscopic ultrasonography is recommended to avoid overestimating the extent of tumor invasion and the resultant aggressive radical surgery. As the developing countries had limited equipment, esophageal resection could be the modality of choice in the treatment.

## Background

Although esophageal leiomyoma is rare, it’s the most common benign submucosal mesenchymal tumor (SMT) of the esophagus, originates from the cells of the smooth muscle, and form near the two-thirds (60–70%) of all benign tumors of the esophageal [1–3]. It almost appears as a single tumor, and multiple leiomyomas of the esophagus are extremely rare [1, 4]. Since esophageal leiomyoma is generally a slow-growing tumor and the size of the tumor may not change for many years, most affected patients are asymptomatic [2]. Often, a diagnosis of esophageal leiomyoma is made as an incidental finding during routine investigation or screening for upper gastrointestinal (GI) pathology [3]. Endoscopic ultrasonography (EUS) and computerized tomography (CT) are used for the diagnosis of leiomyoma. The diagnosis is difficult when multiple leiomyomas coexist with carcinoma lesions. In cases where a carcinoma overlies a submucosal leiomyoma, there is a possibility of overestimating the extent of tumor invasion, and multiple minute leiomyomas are sometimes misdiagnosed as intramural metastasis [2]. Although esophageal leiomyoma is conventionally treated by surgical removal via open thoracotomy for the tumors in the upper two-thirds of the esophagus, minimally invasive approaches like video-assisted thoracoscopic surgery (VATS) and endoscopic resection are other alternative methods used for enucleation of the tumor [3, 5].

The most common epithelial tumor of the esophagus is the squamous cell carcinoma (SCC) [2]. For the identification of an SCC, an upper GI endoscopy with multiple minimally invasive biopsies should be implemented. To assess the size and extent of the primary tumor and to check for the existence of liver metastases and celiac lymphadenopathy, a CT scan of the thorax and abdomen should be done. A precise preoperative staging leads to a proper selection of the treatment. There are some treatment recommendations proposed: (1) Superficial and limited mucosa disease (less than T1a), could undergo endoscopic resection, (2) Lesions penetrating the submucosa with negative lymph nodes (LNs) (more than T1b) could undergo direct surgical resection with lymphadenectomy, (3) Resectable lesions invading muscularis propria with positive LNs (less than T2N1) could receive neoadjuvant chemoradiation, (4) Locally advanced, unresectable or metastatic disease could receive palliative systemic therapy [6].

The simultaneous occurrence of an overlying epithelial lesion (SCC) and a mesenchymal lesion (leiomyoma) is very rare. This study aims to show a case operated due to SCC of esophagus that was postoperatively diagnosed with coexistent esophageal leiomyoma and give a clear overview of the existing literature on it.

## Case presentation

The patient was a 41-year-old woman who was first seen at Shahid Mofateh Clinic affiliated to Yasuj University of medical sciences in 2019 with complaints of having progressive dysphagia to solid foods, and a ten-kilograms weight loss for the preceding 6 months (Weight = 50.5, BMI = 19.77); the patient also had anorexia, decreased intake, and general fatigue and lethargy as accompanying symptoms, but the patient had no nausea and vomiting. Physical examination findings were typically normal. There was no neck (laterocervical or supraclavicular) lymphadenopathy and no hepatomegaly. In past medical and drug history, the patient had asthma and used fexofenadine and salbutamol spray. There was no family history of malignancy in her parents and siblings. The patient did not use tobacco, smoke cigarette and drink alcohol. Laboratory values were all within normal limits.

**Fig. 1:**
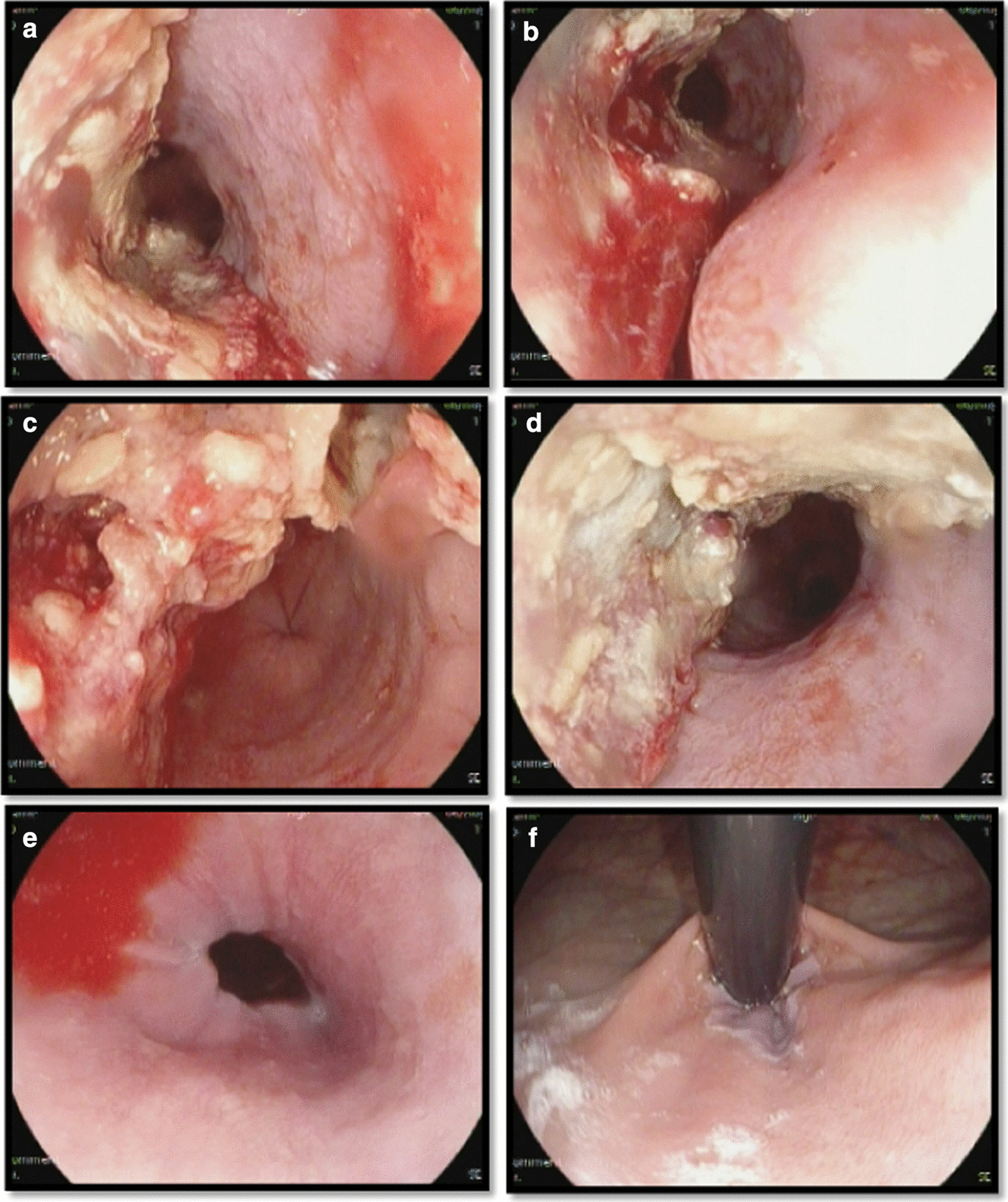
Upper GI endoscopy: large fungating and ulcerative mass in middle (**a** and **b**) and lower third (**c** and **d**) of esophagus, Z-line (**e**), Cardia (**f**)

The patient underwent upper GI endoscopy. In esophagus, the upper esophageal sphincter, cricopharyngeus and upper third of esophagus were normal. A large fungating and ulcerative mass was found in the middle and the lower third of esophagus, 25–33 cm from the upper incisors. The Z line was normal (Fig. 1). In the stomach, the cardia (retro-vision maneuver), fundus, body and antrum were all normal. In the duodenum, the bulb and 2nd part were normal. The patient underwent an incisional biopsy and was found to have poorly differentiated squamous cell carcinoma in pathology evaluation.

**Fig. 2:**
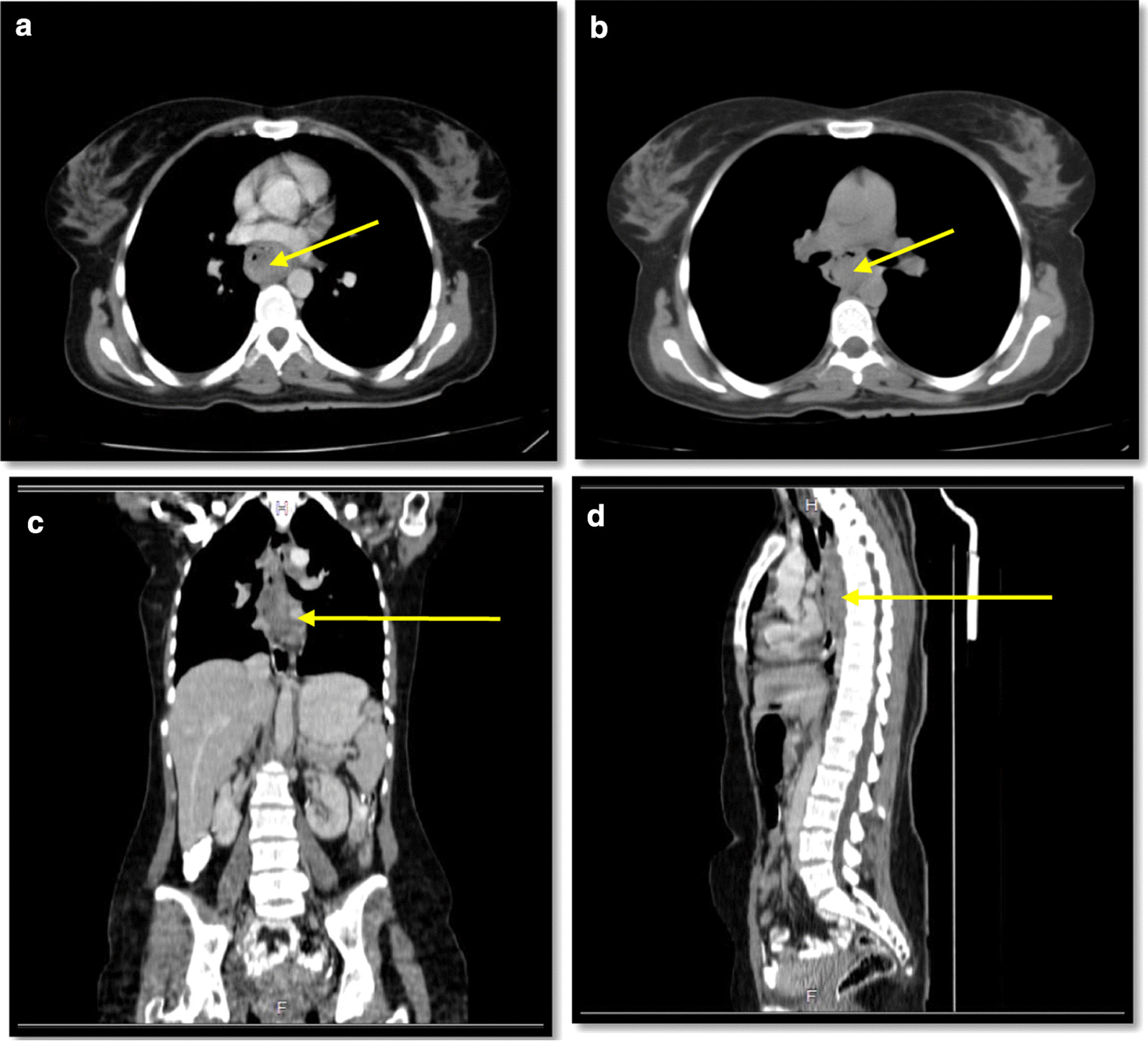
Spiral chest CT scan with and IV contrast (yellow arrows show the tumor); **a** axial view with IV contrast; **b** axial view without contrast; **c** coronal view with IV contrast; **d** sagittal View with IV contrast

**Fig. 3:**
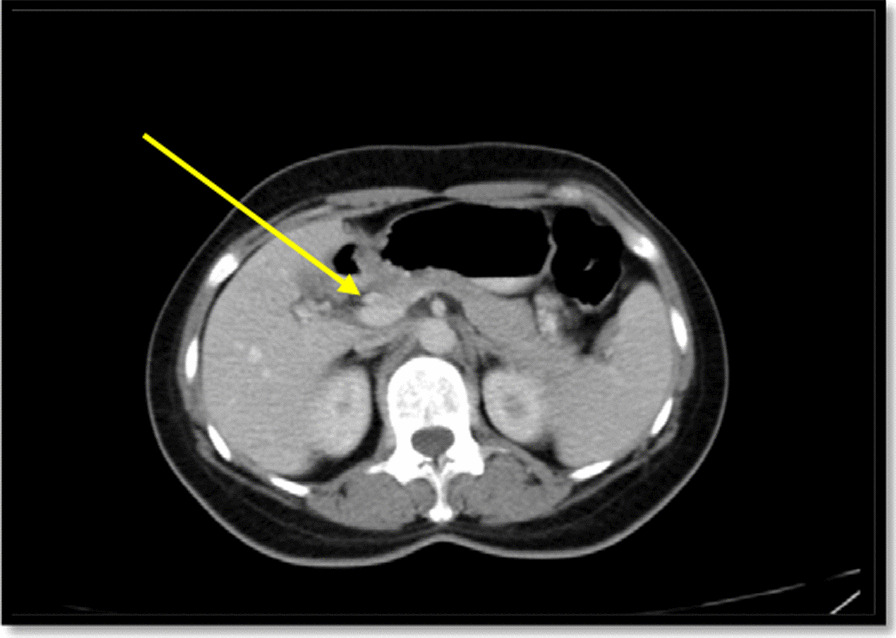
Spiral abdomen and pelvic CT scan: a lymph node within gastrohepatic ligament shown with a yellow arrow

In spiral chest CT scan with and without intravenous (IV) contrast, obtained axial images, represented a 29 × 18 mm soft tissue fullness at middle to distal esophagus (at the level of the carina to the level of the main pulmonary artery), which was suggestive for a tumoral lesion. No lung metastasis was found. In spiral abdomen and pelvic CT scan with oral and IV contrast, there was a 12 × 9 mm LN within gastrohepatic ligament (Fig. 2). In the abdomen and pelvic sonography, there was a 10 × 9 mm hypoechoic lesion between the left liver lobe and greater curvature which was suggestive of lymphadenopathy (LAP) (Fig. 3.).

**Fig. 4:**
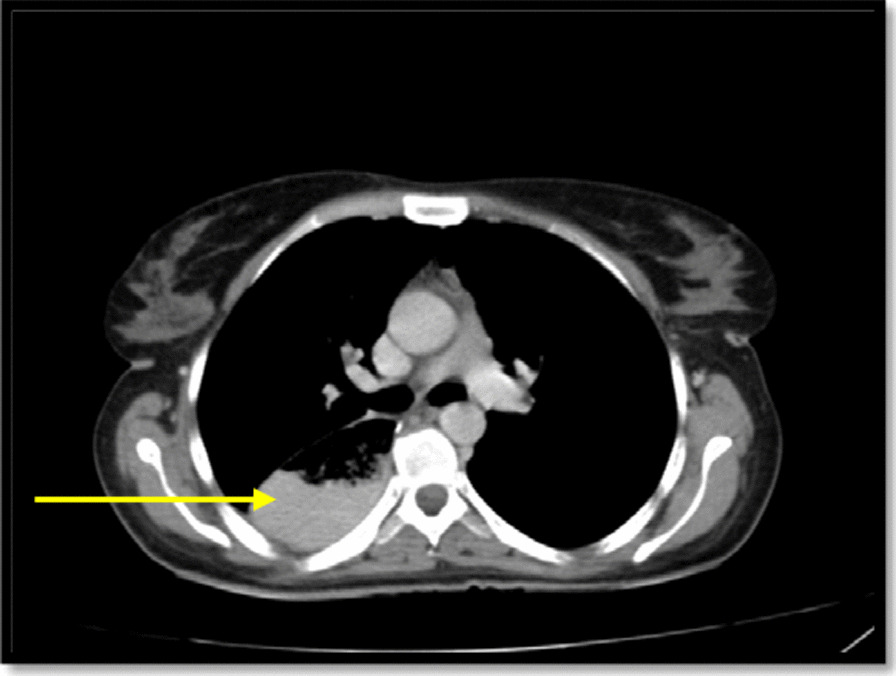
Spiral chest CT scan: pulled-up stomach at the right paraspinal region shown with a yellow arrow

Afterward a multidisciplinary team decision-making meeting (weekly general surgery grand round at the department of surgery, Shahid Beheshti Hospital, Yasuj University of Medical Sciences) was held, and it was decided that the patient would receive 3 sessions of chemotherapy without radiotherapy before undergoing the operation. Then, the patient underwent three field esophagectomy (McKeown) including laparotomy, right thoracotomy, and gastric pull-up with cervical anastomosis including extended thoracotomy, esophagectomy, gastric pull-up, pyloroplasty, cervical esophagostomy, jejunostomy, and chest tube insertion. An abdominal LN at the gastrohepatic ligament was also resected. The postoperative course was uncomplicated. There was no evidence of the leak at the anastomotic site by administration of methylene blue dye on the fifth postoperative day and then nasogastric tube was removed and the patient was told to start drinking liquids. The patient advanced to a regular diet by the tenth postoperative day. She was discharged from the hospital on the 14th day of admission. At a follow-up visit of 1 week and then 2 weeks after discharge from the hospital she was feeling well. The patient’s condition after surgery came back to her normal habitual life within 3 months. The patient had no complaint at the 9th month follow-up visit after surgery. A spiral chest and abdomen CT scan was done and showed a pulled-up stomach, filled with fluid and air at the right paraspinal region (Fig. 4). In the last follow-up, the patient had no complaints and said that she ate a satisfying meal recently, and that she is optimist about the future.

**Fig. 5:**
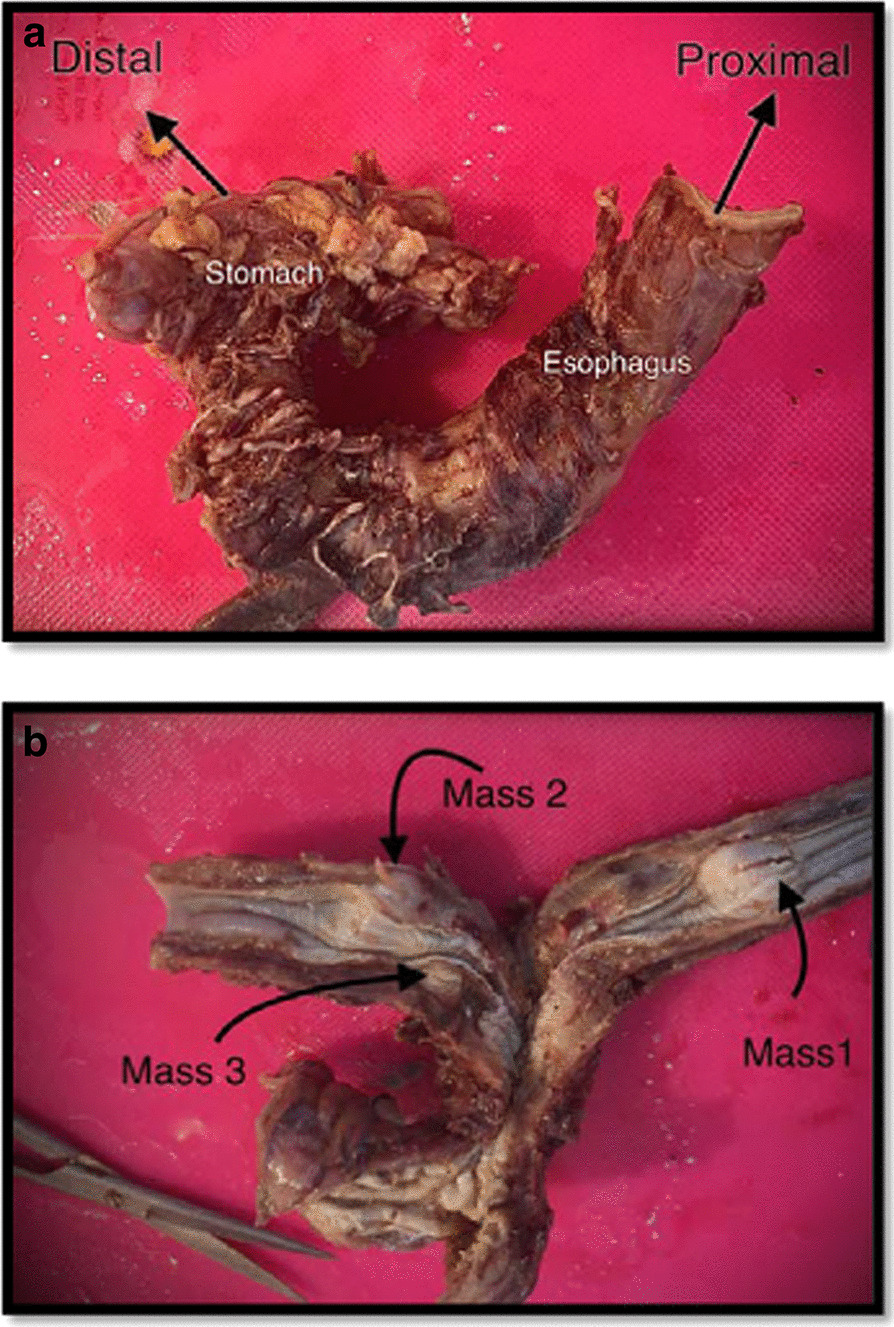
**a** Gross pathology of the specimen, **b** Three leiomyomae in gross pathology of the specimen

**Fig. 6:**
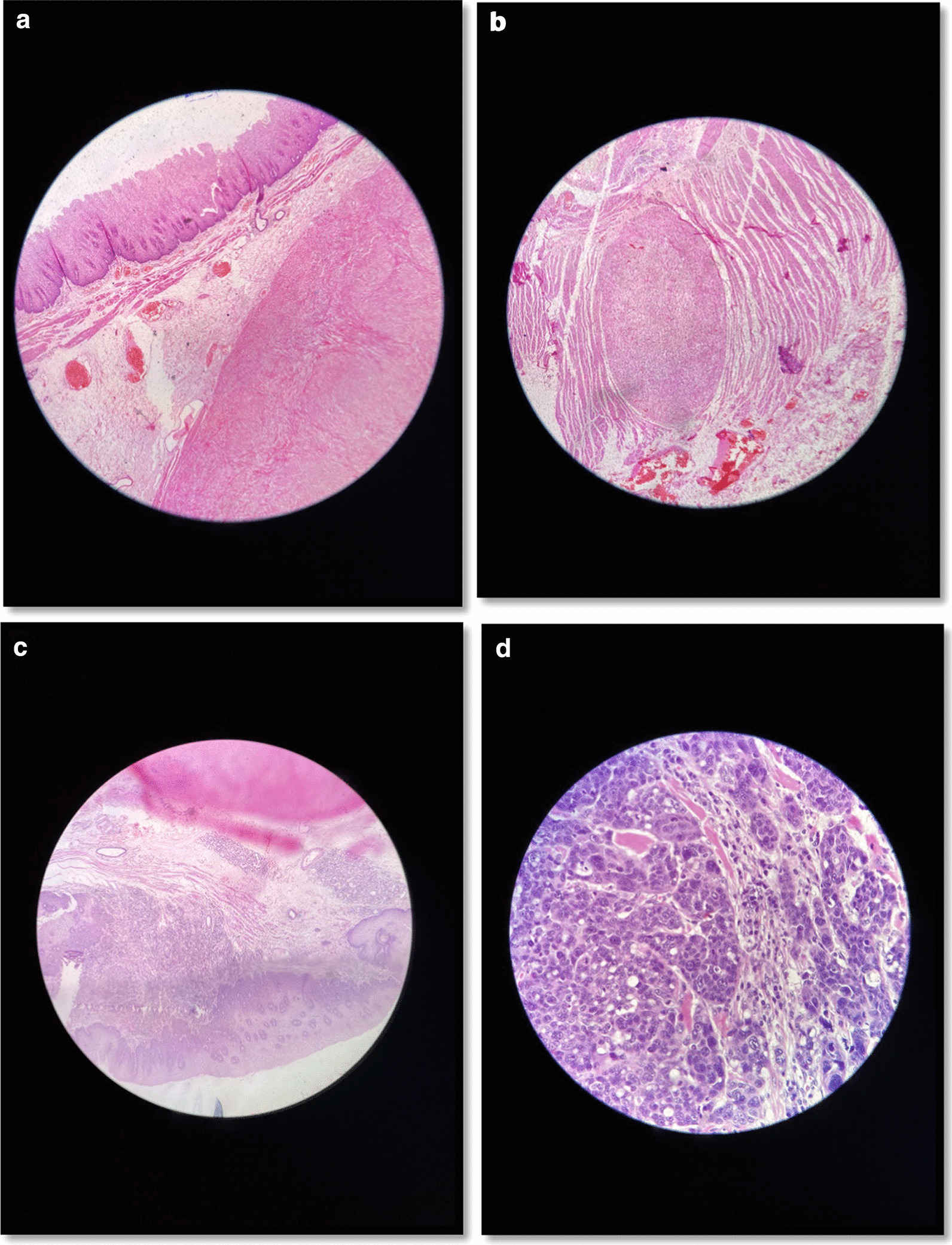
**a** A leiomyoma with normal overlying epithelium, **b** A small separated leiomyoma, **c** Overlying submucosal SCC on a leiomyoma, **d** SCC residual tumor cell nests

The specimen was sent in formalin for pathology and immunohistochemistry evaluation. The received specimen was consisted of the esophagus and a segment of the stomach. The resected esophagus was 12 cm in length and 3 cm in the greatest diameter. The adventitial surface was congested. On opening, there were four intramural creamy round firm masses measuring from 1 to 2 cm. No perforation site was found. Proximal and distal margins were not grossly involved. The resected segment of stomach was 8 cm in greater curvature and 6 cm in lesser curvature and showed no mass (Fig. 5). Multiple LNs were identified measuring from 0.5 to 1 cm in diameter. Pathological evaluation was done, and the patient had small groups of residual poorly differentiated squamous cell carcinoma and also multiple leiomyomas (four). A leiomyoma was coexisted with an invading overlying SCC (Fig. 6). There were 5 isolated LNs within the pathology specimen, of which two were involved with the tumor, but pathology did not show any sign of metastasis in the LNs resected at the gastrohepatic ligament. Tumor pathology characteristics are summarized and shown in Table 1.

**Table 1:** Tumor pathology and immunohistochemistry characteristics

Tumor type	SCC, poorly differentiated (G3)
Residual tumor size	0.6 cm
Tumor extension	Tumor invaded submucosal (at least)
Proximal margin	Free of tumor
Distal margin	Free of tumor
Radial margin	Free of tumor
Treatment effect	Near complete response, score 1 (single cell and small groups of cancer cells
Lymphovascular invasion	Present
Perineural invasion	Present
Lymph-node invasion	Total: 5, involved: 2; metastatic poorly differentiated squamous cell carcinoma (LN2)
Additional pathologic findings	Multiple leiomyomas, the largest one measuring: 2 cm in diameter
Pathologic staging	ypT1bN1Mx
Immunohistochemistry	Negative for C-KIT and CDX2 proto-oncogenes
	Positive for Desmin and P63 proto-oncogenes
Note	This staging categorizes the extent of tumor actually present at the time of examination (after the chemotherapy). The “y” categorization is not an estimate of tumor before chemotherapy

## Discussion and conclusion

There are several reports of co-existing overlying esophageal SCC and leiomyoma in the literature. In this situation, the esophageal submucosal layer is overlaid by the superficial cancer. This coexistence is very rare, and this is usually detected following surgery. Callanan et al., reported the first case of its kind in 1954 [7]. In this regard, all previously published reports on cases with leiomyoma overlying with esophageal SCC were searched and found on the web and only available full-text original articles were encountered. Accordingly, fifteen cases in fourteen reports were found. These reports were evaluated and summarized in Table 2 according to the originated country and published year of case report, patient’s gender, age, chief complaints, risk factors, comorbid diseases, upper GI endoscopy, EUS, other diagnostic modalities, pathological results, location of tumor, and treatments applied [1, 2, 4, 8–18].

**Table 2:** Summarized previous published reports on leiomyoma with overlying SCC

	Country and year	Gender and age	Chief complaints	Risk factors	Comorbid diseases	Upper GI Endoscopy	EUS	Other diagnostic modalities [1]	Other diagnostic modalities [2]	Pathology	Location	Treatment
1	Japan, 1984 [8]	Male/ 75	Stenotic sensation in the pharyngeal region	Heavy smoking	Not available (NA)	An elevated erosive lesion in the posterior wall of the esophagus	NA	Barium esophagogram: a tumorous shadow,5 cm, middle thoracic esophagus	NA	Leiomyoma with overlying SCC	Middle third of the esophagus	Surgery: blunt dissection of the esophagus without thoracotomy, esophagogastroanastomosis
2	Japan, 1995 [9]	Male/45	Dysphagia	NA	NA	NA	NA	NA	NA	Leiomyoma with overlying SCC	Middle third of the esophagus	Esophageal resection and reconstruction
3	Japan, 1995 [9]	Male/71	Not mentioned, referred for treatment of an esophageal tumor	NA	NA	A mucosal irregularity on the surface of the submucosal tumor	NA	NA	NA	Leiomyoma with overlying SCC	Middle third of esophagus	Esophageal resection and reconstruction
4	Japan, 1995 [10]	Male/52	Asymptomatic	Heavy smoking	Negative	Sessile polypoid lesion at abdominal esophagus	A 9 mm submucosal tumor with smooth contour, leiomyoma	Barium swallow: as health checkup	Double contrast barium esophagogram: semispherical tumor, 1.5 cm	Leiomyoma with overlying poorly differentiated SCC	Distal third of the esophagus	Surgical Resection
5	Japan, 1997 [11]	Male/61	NA	NA	NA	Sessile polypoid lesion with a smooth surface at the esophagus	A hypoechoic tumor just below the epithelial layer	NA	NA	Moderately differentiated SCC on leiomyoma originating from muscularis mucosa	Proximal third of the esophagus	Endoscopic resection by aspiration lumpectomy
6	Japan, 2002 [12]	Male/62	Not mentioned, referred for treatment of an esophageal tumor	NA	NA	A protruding lesion, resembling a submucosal tumor, in the proximal third of the esophagus	A hypoechoic tumor confined to the submucosa with a well demarcated, smooth outline	Chromoendoscopy: non-staining	NA	Moderately differentiated SCC overlying the leiomyoma	Proximal third of the esophagus	Endoscopic mucosal resection (EMR)
7	Japan, 2004 [13]	Male/65	NA	NA	NA	A sessile polypoid lesion with a smooth surface in the proximal third of the esophagus	A hypoechoic tumor, 13 mm in diameter, located in the submucosa, with clear margins and a smooth contour, with the muscularis propria layer intact	Chromoendoscopy: non- staining	NA	Leiomyoma with overlying poorly differentiated SCC	Proximal third of the esophagus	subtotal esophagectomy with a three-field (cervical, mediastinal and abdominal) lymph node dissection and esophagogastrostomy
8	Japan, 2006 [2]	Male/71	Firstly, presented with a cervical mass	NA	Malignant lymphoma of the thyroid	A superficial ulcerative tumor [24 mm in diameter] in the lower third of the esophagus, overlying the smaller leiomyoma	Cancer overlaid the smaller leiomyoma and invaded the submucosal layer	Barium swallow Esophagogram: two smoothly rounded defects	Chromoendoscopy: non-staining	Squamous cell carcinoma in situ overlying a leiomyoma, separated multiple leiomyomas	Distal third of the esophagus	Surgery: esophagectomy, gastric pull-up reconstruction, cervical anastomosis via abdominal, left cervical and right thoracotomy incisions
9	Japan, 2008 [14]	Male/61	Mild epigastralgia	NA	NA	A submucosal tumor with a smooth and mildly red surface mucosa in the proximal third of the esophagus	A hypoechoic lesion originating from the muscularis mucosae with a clear margin	Chromoendoscopy with iodine staining: non-staining area	NA	Leiomyoma with overlying SCC	Proximal third of the esophagus	Endoscopic submucosal dissection (ESD)
10	Iran, 2009 [15]	Male/47	Progressive dysphagia and epigastric pain and about 20 kg weight loss	NA	NA	A depressed area with uneven and irregular border	NA	NA	NA	Several leiomyomas, the largest of which was covered by malignant SCC	NA	Laparotomy, transhiatal esophagectomy, cervical esophagastrostomy (Orringer), and bilateral chest tube insertion
11	Japan, 2009 [16]	Male/65	Asymptomatic	Heavy smoking and alcohol consumption	Negative	An esophageal tumor in the middle thoracic esophagus	Hypoechoic tumor in the muscularis mucosae covered with intraepithelial layer indicative of leiomyoma	Chromoendoscopy with iodine staining: non-staining area	Magnifying endoscopy with narrow band imaging (NBI): dilated and elongated intrapapillary capillary loop (IPCL), indicated intraepithelial carcinoma	Leiomyoma with overlying SCC	Middle third of the esophagus	ESD
12	Turkey 2012 [4]	Male/44	Weight loss and dysphagia for two month	Negative	Negative	A tumoral mass with a partially irregular surface in the distal third of the esophagus	NA	CT scan of the thorax and abdomen: minimally diffuse esophageal wall enlargement in a 5 cm segment of the distal esophagus with no lymph node involvement or distant metastasis	NA	SCC infiltrated to the submucosa and two separated leiomyoma along with five reactive and three metastatic lymph nodules	Distal third of the esophagus	Distal esophagectomy and an esophagogastrostomy together with a laparotomy and thoracotomy (Ivor-Lewis approach)
13	Japan, 2013 [17]	Female/75	Esophageal tumor at health checkup	NA	Hyperglycemia and osteoporosis	A submucosal tumor, measuring 5 × 4 mm in diameter, 20 cm from the incisors	NA	NA	NA	Coexistence of esophageal SCC in situ (high-grade intraepithelial neoplasia) overlying leiomyoma	20cm from the incisors (proximal third of the esophagus)	EMR
14	South Korea, 2014 [19]	Male/53	Esophageal tumor at health checkup	NA	NA	An elevated mass, 1.8 cm in diameter, with irregular central small erosion in the upper thoracic esophagus	NA	Chromoendoscopy with iodine staining: non-staining area	NA	Squamous cell carcinoma in situ overlying submucosal leiomyoma	Proximal third of the esophagus	Subtotal esophagectomy
15	South Korea, 2015 [1]	Male/66	Esophageal tumor at health checkup	NA	NA	A protruding mass that measured 1 × 1 cm in diameter and was located 25 cm from the upper incisors	A hypoechoic and homogeneous lesion that originated from the muscularis mucosa (MM) below the epithelial layer	Chromoendoscopy with iodine staining: non-staining area	NA	Squamous cell carcinoma in situ overlying submucosal leiomyoma	Middle third of the esophagus	ESD
16	Iran, 2020 (current study)	Female/41	Progressive dysphagia and significant weight loss for the preceding 6 months	Negative	Negative	A large fungating and ulcerative mass, middle and lower third of esophagus, 25–33 cm from the upper incisors	NA	Chest CT scan: a 29*18 mm soft tissue fullness at middle to distal esophagus, suggestive for tumoral lesion	NA	Multiple leiomyomas, SCC overlying a leiomyoma	Middle and distal third of the esophagus	Extended thoracotomy, esophagectomy, gastric pull-up, pyloroplasty, cervical esophagostomy (Orringer)

Of the previous published reports, 10 reports were from Japan, 2 reports were from South Korea, and one report from Iran and Turkey each. It shows that this pathology is much more common in the East Asia, especially in Japan. There was only one female patient previously reported, so our case was the second one. This displays the male majority in this concern. Ishida et al. reported a male to female ratio of 4:1 in patients with SCC overlying leiomyoma [16]. Mean age was 59.62 ± 9.46 years (minimum 41 and maximum 75 years). Our case was the youngest patient in all, as the youngest patient was previously from Turkey with an age of 44 years [4].

In chief complaints, there were 5 cases with dysphagia or stenotic sensation in the pharyngeal region, 3 cases with significant weight loss, 3 cases with esophageal tumor which were found at the routine health checkups, and one patient with epigastric pain. From the reports in which risk factors were mentioned, heavy smoking was seen in three cases. Comorbid diseases were found negative in 4 cases, and malignant lymphoma of the thyroid and hyperglycemia and osteoporosis were found in one case each. Upper GI endoscopy showed an elevated or protruding lesion in 5 cases, a sessile polypoid lesion in 3 cases, and a submucosal tumor in 3 cases. Chromoendoscopy with iodine staining was done in 7 cases and showed a non-staining area in all. EUS was done in 8 cases and showed a hypoechoic homogeneous submucosal tumor with a well demarcated and clear margin in the muscularis mucosae (MM) covered with the intraepithelial layer indicative of leiomyoma in most of the cases. Barium esophagogram study was done in 4 cases. In pathological evaluation, squamous cell carcinoma in situ (high-grade intraepithelial neoplasia) was seen in 4 cases, poorly differentiated SCC in 2, moderately differentiated SCC in 2, and malignant SCC in one case. More than one leiomyoma was seen in 3 cases. According to the location of the lesions, proximal, middle and distal third of the esophagus accounted for 6, 6 and 4 cases each  respectively. Surgery (esophageal resection) was done in 10 cases, and this is comparable with endoscopic removal which was done in 5 cases.

There are two types of coexistence: (1) the carcinoma covers the benign tumor in the overlying type, and (2) completely separated lesions elucidated through or after the esophagectomy for esophageal SCC [2, 12, 19]. In our case study, the two kinds of coexistence were present and this result was compatible with the results of two other studies done by Iwaya et al. and Geramizadeh et al. [2, 15]. Two concepts might be important in the pathogenesis of the disease and carcinoma development over a leiomyoma: (1) A bulging of the leiomyoma into the esophageal lumen may cause the esophageal mucosa to be irritated chronically and predispose it to dysplasia; (2) The underlying leiomyoma itself may prevent the overlying SCC from spreading and also a deep invasion [2, 13, 15].

For the differentiating esophageal carcinoma and leiomyoma, an upper GI endoscopy is usually used, but as the esophageal leiomyomas are submucosal lesions, it may not always lead to a precise diagnosis. Detection and management of these tumors could be done by the use of EUS by revealing the five-layered structure of the gastrointestinal wall as well as by enabling exact localization and origin, tumor margin, echogenic pattern, and exact size measurement [4, 5]; but unfortunately we didn’t have EUS in our hospital, so we were unaware about coexistence of any leiomyoma until it had been removed through esophagectomy.

In narrow-band imaging (NBI) endoscopy, optical filters are used which increase absorbance and scattering of light, and this would boost vessels and other structures appearance, and could afford a high tissue contrast throughout the endoscopy. So with the means of magnifying endoscopes and chromoendoscopy, description of histological tissue and assessment of esophageal lesions are now possible [5]; but unfortunately we did not have any of these to help us evaluate the tumor or esophageal mucosa.

In SMTs which are not accompanied by carcinomas, it is important to find out which layer the tumor originates from by the use of EUS. Endoscopic mucosal resection (EMR) is the treatment of choice in a small SMT originating from the muscularis mucosa. Open surgery or thoracoscopic resection is chosen in a large SMT originating from the muscularis propria. In these cases EMR can’t be used because of high risk of complications, such as esophageal perforation and massive bleeding [13]. In our case study, we didn’t have EUS in our hospital, so we were unaware about coexistence of any leiomyoma until it had been removed through esophagectomy.

Locally advanced oesophageal cancer is usually treated with neoadjuvant therapy and then surgery, but it’s still controversial in choosing between neoadjuvant chemoradiotherapy and chemotherapy, and moreover, there are no benefits according to the patients’ survival in using neoadjuvant chemotherapy or neoadjuvant chemoradiotherapy in these patients. This result was understood from a study done by von Döbeln et al., in which a greater tumor tissue reaction in neoadjuvant chemoradiotherapy recipients was found, contrary to no increasing change in patients’ survival. He concluded that the radiotherapy should not be added to the neoadjuvant chemotherapy on a routine basis in the patients with resectable esophageal cancer, and should be used in selected patients [20]. In another study done by Visser et al., no differences between neoadjuvant chemotherapy and neoadjuvant chemoradiotherapy were seen in postoperative complications and in-hospital mortality in patients treated for esophageal adenocarcinoma [21]. In the current study, preoperative chemotherapy without radiotherapy was done, which was actually a decision made in a multidisciplinary team due to the facts mentioned above.

To assess an effective esophagectomy with proper LN dissection, the number of LNs retrieved are counted as a quality indicator [22, 23]. In a new study done by van der Werf et al., the number of LNs harvested was associated with more precise staging, but did not have an influence on patient’s survival or morbidity and mortality [24]. The number of LNs retrieved is related to the several patient and disease features, including preoperative weight loss, low Charlson comorbidity score, higher clinical N stage, using neoadjuvant therapy, surgical approach (transthoracic or transhiatal), year of resection, and hospital volume [22, 23]. Neoadjuvant chemoradiotherapy would cause the tumor and lymph node down-staging and result in more resections with negative margins and lymph nodes [23, 25]. In our study only five LNs were harvested, and as the patient received three sessions of chemotherapy before the operation, we could propose that the few numbers of the LNs harvested in this study, could be due this fact.

To sum up it can be concluded that (1) It must be kept in mind that esophageal carcinomas may coexist with leiomyomas, (2) Preexisting benign tumors may have played an important role in the development of carcinoma by inducing constant stimulation of the overlying mucosa, (3) EUS is recommended to avoid overestimating the extent of tumor invasion and the resultant aggressive radical surgery (4) As the developing countries had limited equipment, esophageal resection could be the modality of choice in the treatment.

## Data Availability

The datasets used and/or analyzed during the current study are available from the corresponding author on reasonable request.

## Abbreviations

NA: Not available
SMT: Submucosal mesenchymal tumor
LN: Lymph node
EUS: Endoscopic ultrasonography
CT: Computerized tomography
GI: Gastrointestinal
VATS: Video-assisted thoracoscopic surgery
SCC: Squamous cell carcinoma
IV: Intravenous
MM: Muscularis mucosae
ESD: Endoscopic submucosal dissection
EMR: Endoscopic mucosal resection
NBI: Narrow band imaging
